# A Response Surface Model Exploration of Dosing Strategies in Gastrointestinal Endoscopies Using Midazolam and Opioids

**DOI:** 10.1097/MD.0000000000003520

**Published:** 2016-06-10

**Authors:** Jing-Yang Liou, Chien-Kun Ting, Ming-Chih Hou, Mei-Yung Tsou

**Affiliations:** From the Department of Anesthesiology, Taipei Veterans General Hospital (J-YL, C-KT, M-YT), National Yang-Ming University and School of Medicine (C-KT, M-CH, M-YT), and Center for Diagnostic and Treatment Endoscopy, Taipei Veterans General Hospital, Taipei, Taiwan, ROC (M-CH).

## Abstract

Classical midazolam–opioid combination for gastrointestinal endoscopy sedation has been adopted for decades. Dosing regimens have been studied but most require fixed dosing intervals. We intend to use a sophisticated pharmacodynamic tool, response surface model (RSM), to simulate sedation using different regimens. RSM can predict patient's response during different phases of the examination and predict patient's wake-up time with precision and without the need for fixed dosing intervals. We believe it will aid physicians in guiding their dosing strategy and timing.

The study is divided into 2 parts. The first part is the full Greco RSMs development for 3 distinct phases: esophagogastroduodenoscopy (EGD), colonoscopy, and intersession (the time lapse between procedures). Observer's Assessment of Alertness Score (OAA/S) is used to assess patient response. The second part simulates 6 regimens with different characteristics using the RSMs: midazolam only, balanced midazolam and opioids, high-dose opioids and midazolam, low-dose midazolam with high-dose opioids, high-dose midazolam and low-dose opioids, and finally midazolam with continuous opioid infusion. Loss of response at 95% probability for adequate anesthesia during examination and return of consciousness at 50% probability during intersession was selected for simulation purposes.

The average age of the patient population is 49.3 years. Mean BMI is 21.9 ± 2.3 kg/m^2^. About 56.7% were females and none received prior abdominal surgery. The cecal intubation rate was 100%. Only 1 patient (3%) developed temporary hypoxemia, which was promptly managed with simple measures. The RSMs for each phase showed significant synergy between midazolam and alfentanil. The balanced midazolam and alfentanil combination provided adequate anesthesia and most rapid return of consciousness. The awakening time from the final drug bolus was 7.4 minutes during EGD and colonoscopy stimulation, and 9.1 minutes during EGD simulation.

Simulation of regimens with different characteristics gives insights on dosing strategies. A balanced midazolam–alfentanil regimen is adequate in providing good anesthetic depth and most rapid return of consciousness. We believe with the aid of our RSM, clinicians can perform sedation with more flexibility and precision.

## INTRODUCTION

Gastrointestinal endoscopy sedation should be characterized by rapid induction of sufficient amount of anesthetics and rapid awakening. Optimal dosing regimen selection in literatures focused on the quality of sedation, occurrence of side effects, and time to awakening among different anesthetic individuals.^1–4^ Many studies indicate that a single hypnotic agent for gastrointestinal endoscopy does not provide a good analgesia and hypnosis at the same time.^1,5^ Single-agent regimens generally require a larger amount of drug infused and therefore side effects such as respiratory depression are likely to appear. Good instrumentation conditions as well as less drug side effects can be achieved by combining an opioid and a hypnotic agent.^1^ Recent American and European guidelines focused on the use of propofol^6–8^ and works regarding the use of propofol have been vigorously done.^9,10^ However, propofol has a narrower therapeutic window and may increase the chance of hypotension or respiratory depression that requires intervention as compared with the use of midazolam and alfentanil.^11^ There are significantly less works emphasizing the dosing strategies using a benzodiazepine and an opioid, probably because they possess more complicated pharmacokinetic properties.^12^ Alfentanil is a good opioid choice for gastrointestinal endoscopies because of its quick onset time (1–2 min after intravenous injection), short t_1/2__ke0_ resembling thiopental,^13^ and short duration of effect lasting approximately 10 minutes.^13^ There are also interesting works done by Short et al^14^ indicating stronger synergism between midazolam and alfentanil than propofol and alfentanil.^15,16^ Lower potency opioids such as meperidine, tramadol, or morphine or adjuvants such as clonidine are generally not used during outpatient procedures. The longer duration of action for these drugs may raise concerns about respiratory function or blood pressure when discharging the patient.

Giving 2 drugs simultaneously requires the knowledge of how they interact with one another. Response surface model (RSM) is a sophisticated pharmacodynamic tool for analyzing drug interaction and responses for multiple drugs.^17,18^ A 2-drug model is usually used because triple or more drug interactions increase model complexity significantly and is less comprehensible graphically. A surface will encompass the complete set of isobolograms, concentration–effect curves, and curve shift effects. The RSMs are most often used clinically to predict wake up time,^1,18,19^ tolerance to stimuli,^20–22^ or occurrence of side effects.^1,23^ We can help define the optimal dosing regimen by analyzing different but relevant surfaces simultaneously. Another feature that makes RSMs stand out from other models is that it does not require a fixed dosing interval and can cope with very variable examination time.

This study is designed to tackle several issues. First, traditional dosing regimen studies require a fixed dosing timetable and examination time. Second, very little work has been done for the benzodiazepine-opioid RSM. Our study is divided into 2 parts. The first part of our study involves building the model for simulation. The second part of the study deals with different midazolam and opioid simulations through RSM, which was developed in the first part, to rationally delineate dosing strategies for gastrointestinal endoscopy.

## METHODS

### Patient Population, Management, and Model Building

Patients between 18 and 65 years old, American Society of Anesthesiologists (ASA) Physical status I ∼II who underwent EGD (esophagogastroduodenoscopy) and colonoscopy as a single-stage procedure, were chart-reviewed. Those with documented verbal communication impairment, cerebrovascular diseases, incomplete records, or a history of sedative, opioid, or chronic alcohol use were excluded. Requirement for written informed consent was waived by the Institutional Review Board (IRB 2014-12-001BC) of Taipei Veterans General Hospital. After setting up the patient on standard anesthetic care monitoring, continuous 3 L/minute supplemental oxygen was given through nasal cannula to maintain oxygen saturation greater than 90%. One anesthesiologist performed sedation using midazolam and alfentanil. The induction dose of midazolam and alfentanil was 0.03∼0.04 and 6∼9 mg/kg, respectively. A single gastroenterologist performed the examinations.

The examinations were divided into 3 distinct phases for model development: EGD, colonoscopy, and intersession (the time lapse between the procedures). Patient responses were evaluated with the Observer Assessment of Alertness/Score (OAA/S) (Table 1).^24,25^ We define OAA/S < 2 as loss of response (LOR) in EGD and colonoscopy, while OAA/S < 4 indicates LOR during intersession. The plasma drug concentrations are calculated using TIVAtrainer (Version 9.1, Build 5, Euro SIVA, Netherlands). Opioid concentrations are converted to alfentanil equivalents using a potency ratio (fentanyl: alfentanil: remifentanil = 1: 0.0625: 1.2).^17^ The Greco RSM construct is selected for model building (Equation 1)^26^ 
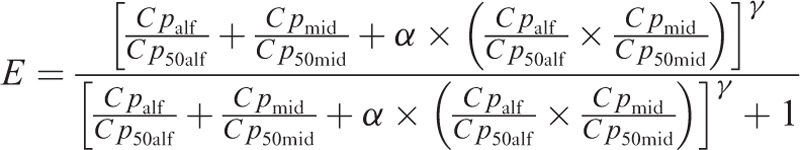


**TABLE 1:**
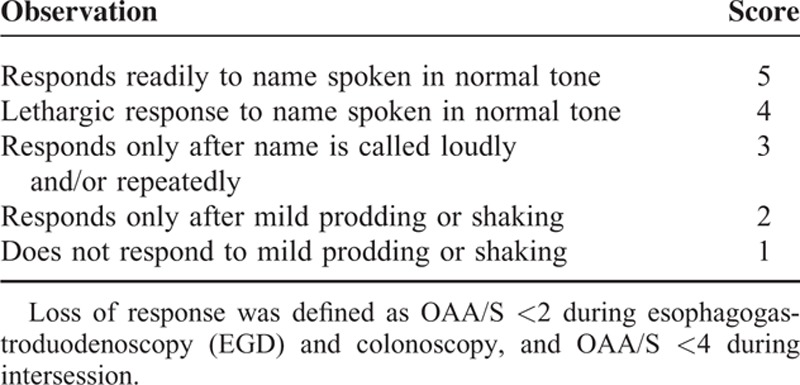
Observer's Assessment of Alertness/Sedation (OAA/S) Scale^24^

where E is the probability of LOR and ranges from 0 to 1. Plasma concentration of the drug is expressed as Cp. Cp_50_ is the value in which the concentration of the drug is required to exert 50% maximal effect alone. The parameter α is the interaction relationship between midazolam and alfentanil, and γ is the steepness that describes the descent of the surface. Interaction is synergistic when α > 0, infra-additive when α <0, and additive when α equals 0. The RSM is an extension of the Hill equation. The resulting 3-dimensional surface theoretically holds the entire drug interaction relationship for the given endpoint, in this case LOR. Single or multiple isoboles or concentration-effect curves can be extracted from the surface to work with. We used Matlab (R2013a; The MathWorks, Inc., Natick, MA) software to perform model fit and parameter estimation. Bootstrap-based programming was written with 1000 iterations for model stability. Parameters for each of the 3 phases are obtained for simulation analysis.

#### Regimen Identification and Simulation

Dosing regimens are searched in PUBMED using the keywords: midazolam, alfentanil, fentanyl, remifentanil, colonoscopy, and gastrointestinal endoscopy. Studies with insufficient dosing details, pediatric patients, geriatric patients, or use of premedications were excluded. Regimens with specific characteristics are sought: midazolam only, balanced midazolam and opioids, high-dose opioids with high-dose midazolam, low-dose midazolam with high-dose opioids, high-dose midazolam and low-dose opioids, and finally midazolam with continuous opioid infusion. Pure opioid regimens were not candidates because our endpoint requires the patient to be unresponsive. Two sets of simulation will be performed, EGD alone and EGD immediately followed by colonoscopy. The 50% probability is the targeted value for model-predicted return of consciousness. Adequate anesthesia occurs at our defined 95% probability of LOR. Our main goal is to find an optimal dosing regimen that will provide adequate anesthesia and the most rapid return of consciousness.

## RESULTS

### Patient Population, Drug Dosage, and RSM

Forty patients were enrolled, but in the end only 33 were eligible for model building. Of the excluded ones, 5 had an incomplete record and 2 had known cerebrovascular disorders. The patient demographics are summarized in Table 2. All patients were ASA physical status I or II, and 56.7% were females. No patient received prior abdominal surgery.

**TABLE 2:**
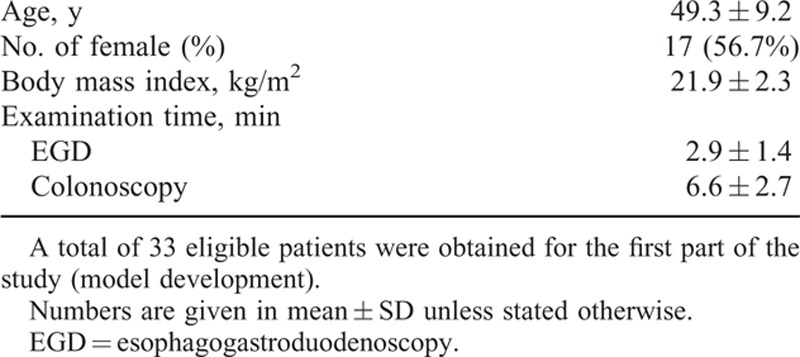
Patient Demographics for Response Surface Model Development

The cecal intubation rate was 100%. During the procedures, only 1 patient developed hypoxemia that required intervention. Pulse oximetry displayed saturation between 80% and 90% for approximately 2 minutes. Chin-lift maneuver with increased oxygen flow through nasal cannula increased the patient's saturation above 90%. There were no subsequent events.

The number of concentration pairs available for pooling and model building were 68, 75, and 75 for EGD, colonoscopy, and intersession, respectively. Total midazolam dosage was 0.049 ± 0.016 and 0.013 ± 0.005 mg/kg for alfentanil. The required model parameters (for Equation 1) are summarized in Table 3. The α for all the models was greater than zero, indicating significant synergy between midazolam and alfentanil. The Cp_50alf_s were all very large and not achievable with normal dosing during these procedures. This is consistent with the fact that opioids do not produce hypnosis well.

**TABLE 3:**
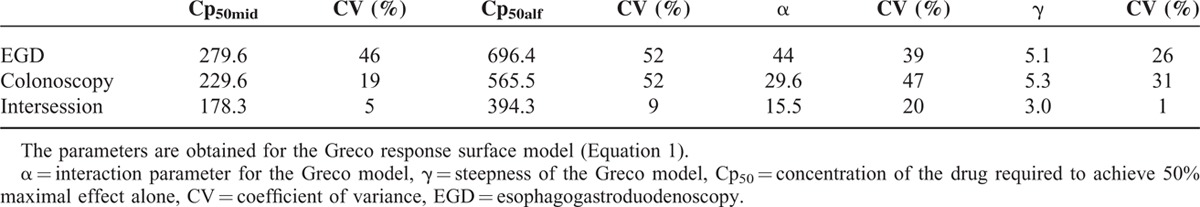
Response Surface Model Parameters

### Simulation Regimens Setup

We used a 49-year-old female who weighted 60 kg and has a body mass index of 21 kg/m^2^ as our simulation patient according to our patient demographics. Slight modifications from the original identified regimens had to be made to define specific dosing intervals and dosages for simulation (Table 4).^27–31^ Study selection is based on the dosing characteristics mentioned previously. Only midazolam is given in regimen 1A, while a balanced dosing mimicking daily practice is 1B. Regimen 2 represents a low opioid–high midazolam combination. Regimen 3 is a high opioid–high midazolam regimen. A low midazolam–high opioid combination is in regimen 4, and lastly midazolam with an opioid infusion scheme is in regimen 5.

**TABLE 4:**
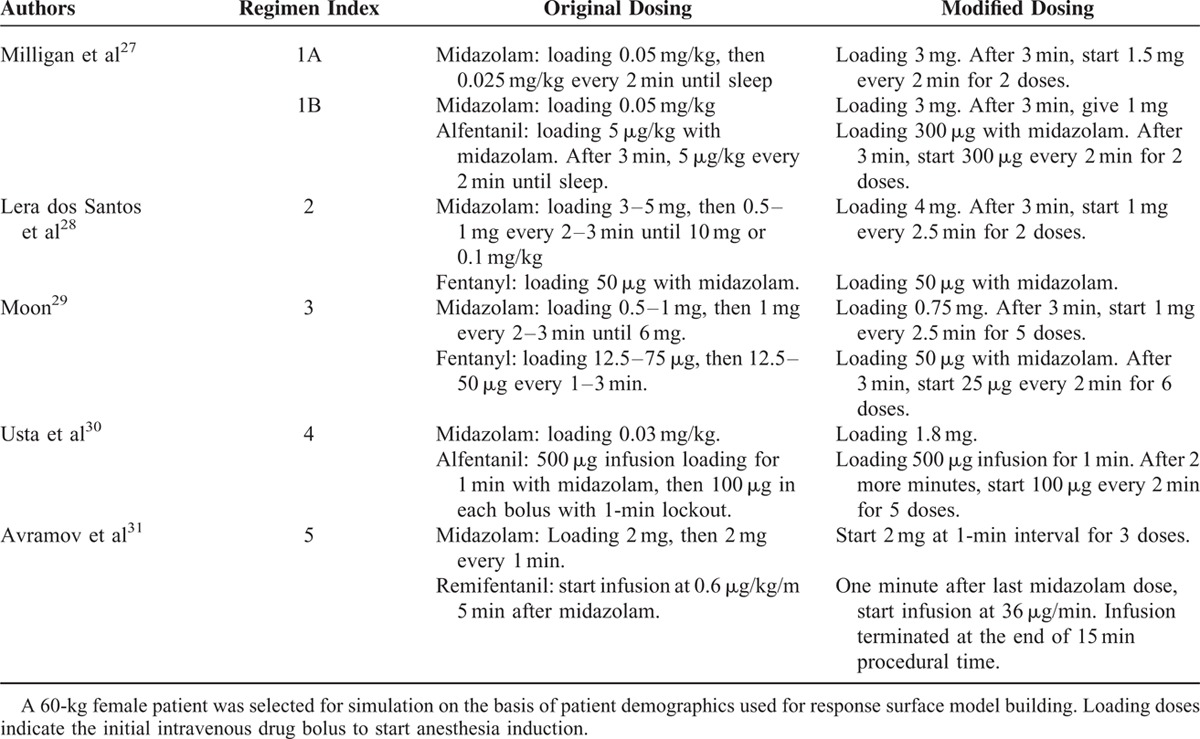
Dosing Regimens for Esophagogastroduedenoscopy and Colonoscopy

We have divided each simulation into 3 periods: 3 minutes of induction time, procedural time (5 min of EGD followed by 10 min of colonoscopy), and a drug washout period of 15 minutes in EGD with colonoscopy and 25 minutes in EGD only. The intersession RSM is applied to the induction and washout phase. The general rule is that midazolam is given at the beginning of the induction because the onset time is approximately 3 minutes^32^ and not given within 3 minutes before the end of procedure. Alfentanil and fentanyl are also not given 3 minutes before the end of procedure. Remifentanil is started at the beginning of procedure rather than during induction because it onsets and offsets very rapidly. Subsequent dosage and time interval were specified by the selected studies. The maximal dosage for each of the drugs during simulation is as follows: 6 mg for midazolam, 1000 μg for alfentanil, and 200 μg for fentanyl.^29^

### EGD and Colonoscopy Simulation

**FIGURE 1:**
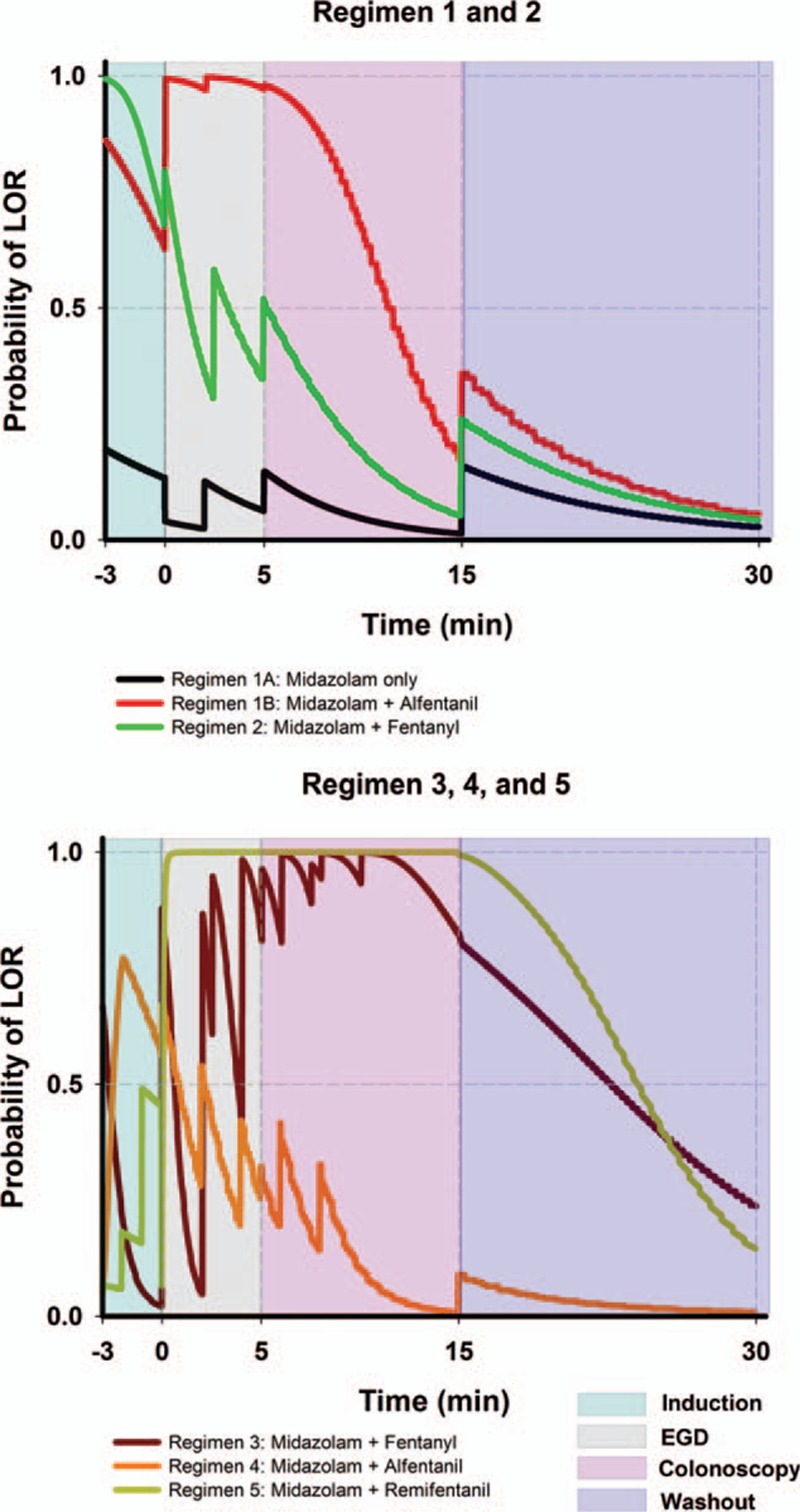
Simulation for esophagogastroduedenoscopy and colonoscopy. The examination time is preceded by a 3-min induction phase and followed by a 15-min washout phase. Induction and washout phase uses the intersession response surface model. Adequate anesthesia is defined as probability reaching 0.95 and wake-up time as 0.5. Opioids are converted to alfentanil equivalents for simulation. EGD = esophagogastroduedenoscopy, LOR = loss of response.

**FIGURE 2:**
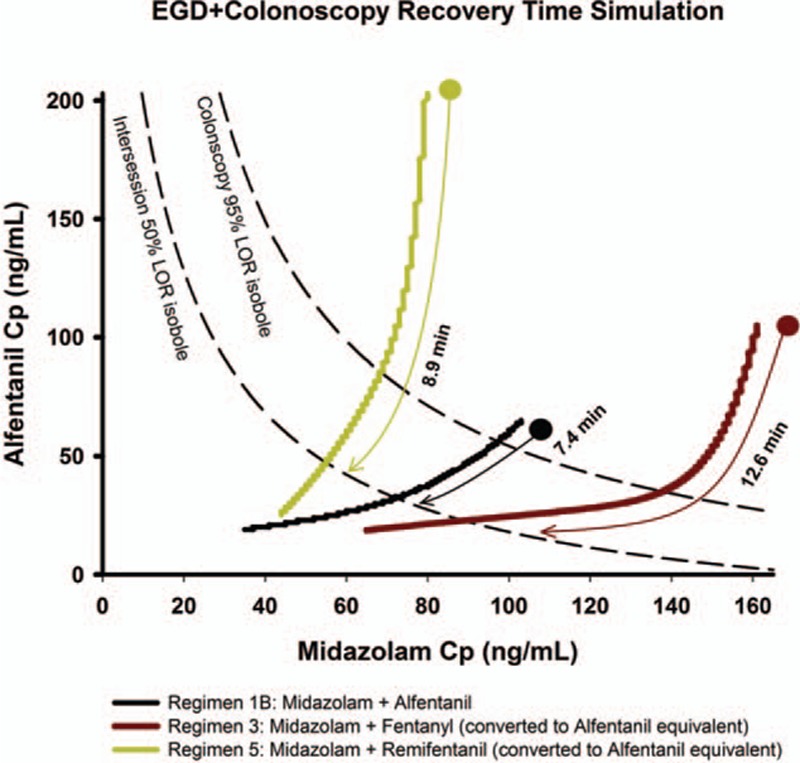
Recovery time simulation of esophagogastroduedenoscopy and colonoscopy. Only regimens that have reached adequate anesthesia are drawn (regimen 1B, 3, and 5). The starting point corresponds to the last bolus medication during simulation, which is usually within the colonoscopy phase. Time to recovery is the estimated time to return of consciousness in a nonpainful state. A good regimen should reach beyond the 95% colonoscopy isobole and return to the intersession 50% isobole rapidly. EGD = esophagogastroduedenoscopy, LOR = loss of response.

The simulation results for the 5 regimens are illustrated in Figure 1. This graph consists of 3 different response surface analyses. They are the induction, EGD, colonoscopy, and washout phase in order from left to right. Saw-like contour of regimens 3 and 4 was a result of multiple drug boluses. Only regimen 1B, 3, and 5 reached our defined depth of adequate anesthesia during the procedures. In regimen 1, return of consciousness should have occurred during the final phases of colonoscopy, which was 2.4 minutes before the washout period. Time to return of consciousness occurred 7.6 minutes into the washout period in regimen 3, and 9.1 minutes in regimen 5. Temporal analysis from 95% LOR to awakening for regimen 1B, 3, and 5 was performed (Figure 2), and regimen 1B had the quickest awakening time of 7.4 minutes from the last bolus.

### EGD-Only Simulation

**FIGURE 3:**
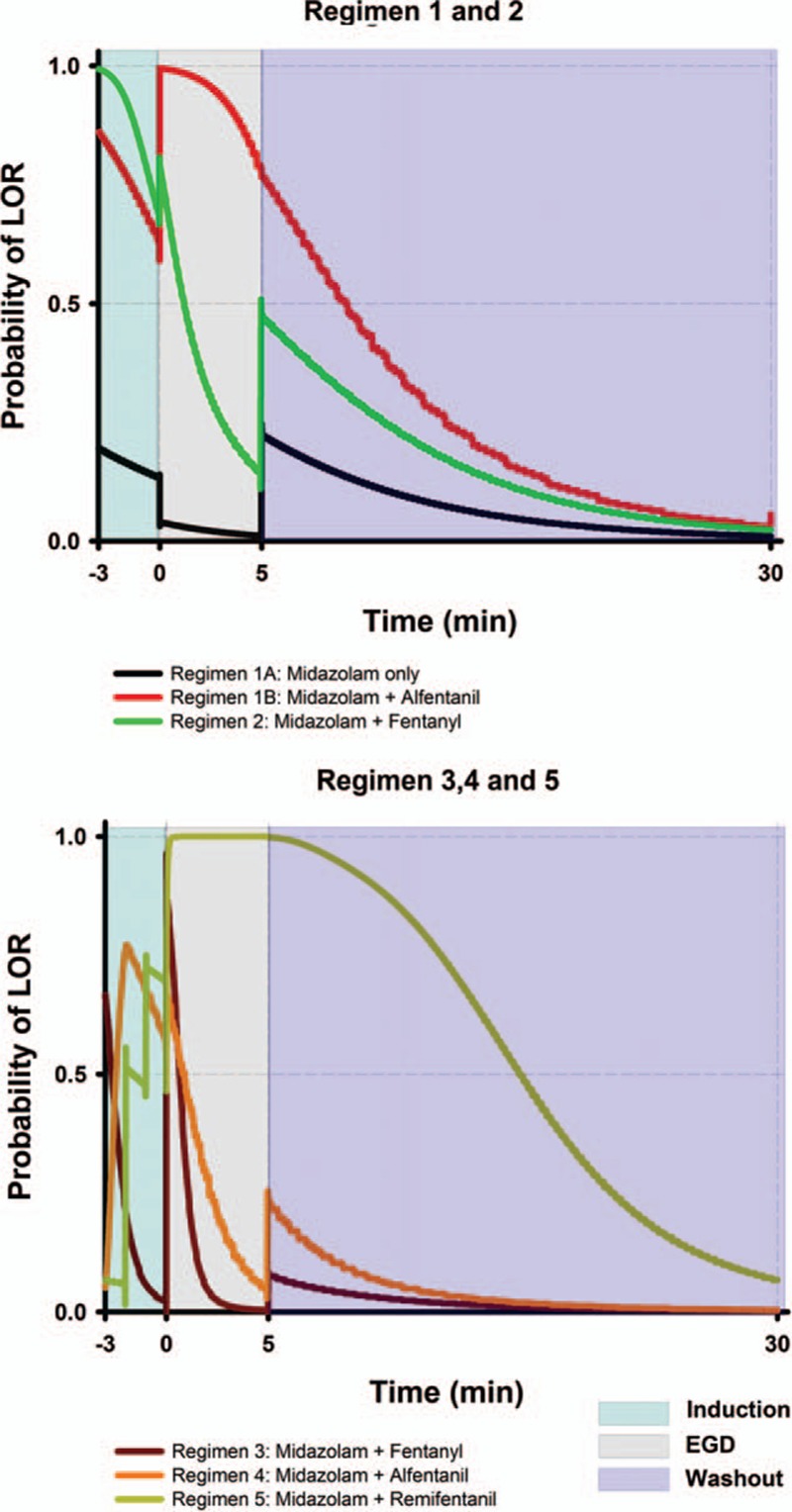
Simulation for esophagogastroduedenoscopy only. The examination time is preceded by a 3-min induction phase and followed by a 25-min washout phase. Induction and washout phase uses the intersession response surface model. Adequate anesthesia is defined as probability reaching 0.95 and wake-up time as 0.5. Opioids are converted to alfentanil equivalents for simulation. LOR = loss of response.

This simulation is done without colonoscopy and further highlights the need for a very rapid increase in anesthetic depth and return of consciousness. Only regimen 1B and 5 reached adequate anesthesia (Figure 3) during EGD. Time to return of consciousness was 4.1 minutes into the washout period for regimen 1B, and 12.3 minutes for regimen 5. Temporal analysis of regimen 1B and 5 also showed earlier return of consciousness with regimen 1B from the last drug bolus.

## DISCUSSION

Our study has graphically illustrated how regimens with different characteristics perform during gastrointestinal procedures. Midazolam and opioids together are capable of providing reasonably rapid return of consciousness, and can be predicted by RSMs. Regimen 1B with balanced midazolam and alfentanil administration consistently resulted in adequate anesthesia and more rapid recovery.

The combination of midazolam and an opioid for procedural sedation is widely used and provide faster return of consciousness than diazepam.^33^ Concurrent administration of a hypnotic agent and an opioid gives better instrumentation condition ^4^ safety and higher patient satisfaction.^34,35^ Bannert et al^36^ reviewed 52,506 colonoscopies and concluded that sedation increased cecal intubation rate in both men and women, but the effect is more pronounced in women. Midazolam–opioid combination reduced the rate of hypoxemia as compared with midazolam alone. A meta-analysis reported hypoxemia in 18% of the patients when midazolam is used alone, and 6% when midazolam is combined with an opioid.^10^ In our patients, only 1 (3%) developed hypoxemia requiring intervention. When compared with propofol, midazolam may result in less hypoxemia events and require less anesthesiologist interventions.^37^

The cecal intubation rate was 100% in our patients and there is a low chance of respiratory depression (3%) during anesthesia. Model built from this population implies that the RSM can facilitate colonoscopies while avoiding unwanted respiratory depression. Hemodynamic stress from endoscopy pain can contribute to myocardial ischemia. Sedation offers additional benefits and a prospective study concluded that sedation with midazolam and propofol may provide protection against stress response.^38^

### EGD and Colonoscopy Simulation

In Figure 1 (and also Figure 3), the sudden increase of probability of LOR is due to bolus medications or a shift from a high-intensity stimulus to a low-intensity stimulus (as from colonoscopy to washout phase). The drop in LOR probability is either due to drug concentration decay or a shift from a low-intensity stimulus to a high-intensity stimulus (as from induction to EGD). We have identified regimen 1B as the better technique. Probability of LOR below 50% near the end of colonoscopy was considered as acceptable because pain is usually minimal near the end of colonoscopy. A study divided colonoscopy into 3-minute segments of initial-, mid-, and final moments and concluded that the final moments were associated with less pain.^35^ During colonoscopy, higher pushing force is at the sigmoid colon and this part of the colon is associated with the highest perforation rate (0.045%).^39^ Another study used magnetic endoscope imaging also reported pain most commonly occurred at sigmoid colon (77%), while the proximal colon was infrequent (4%).^40^ It would seem unnecessary to keep the same anesthetic depth in the second half of colonoscopy as the beginning.

Regimen 1A produced very poor LOR and reflects the fact that sedation with a single agent should be avoided. Many sedation regimens use single agents for comparison, either at low or high doses.^10^ Most hypnotic agents such as propofol and midazolam lack analgesic property. It would require very large doses of hypnotics to cover the pain encountered during endoscopies, along with a higher chance of unwanted side effects and poorer instrumentation conditions.^1,41^ Inadequate anesthetic depth in regimen 2 and regimen 4 implies that neither low opioid–high midazolam or low midazolam–high opioid combinations are regimens of choice.

Regimens 3 and 5 tend to overshoot drug concentration required to produce adequate anesthesia (Figure 2). It takes longer for patients to regain consciousness using these 2 regimens than regimen 1B. Figure 2 deals with time to return of consciousness after the final drug bolus. An estimation of the time patient under anesthesia can be derived from it. This will aid sedation providers in practice.

### EGD-Only Simulation

**FIGURE 4:**
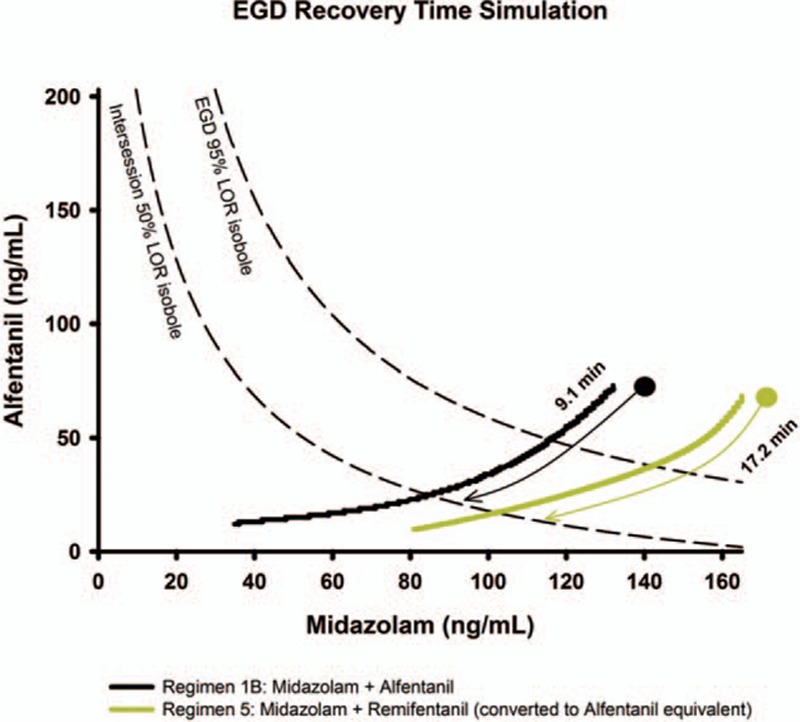
Recovery time simulation of esophagogastroduedenoscopy only. Only regimens that have reached adequate anesthesia are drawn (regimen 1B and 5). The starting point corresponds to the last bolus medication during simulation, which is usually within the colonoscopy phase. Time to recovery is the estimated time to return of consciousness in a nonpainful state. A good regimen should reach beyond the 95% colonoscopy isobole and return to the intersession 50% isobole rapidly. EGD = esophagogastroduedenoscopy, LOR = loss of response.

Regimen 1B has also been identified as the better performing technique, with adequate anesthesia and faster return of consciousness. The total midazolam used was 4 mg and alfentanil was 600 μg. With this dosing, anesthesia is anticipated to last for 9.1 minutes (Figure 4) and well covers the course of EGD.

### Concentration Navigation of the Surface

**FIGURE 5:**
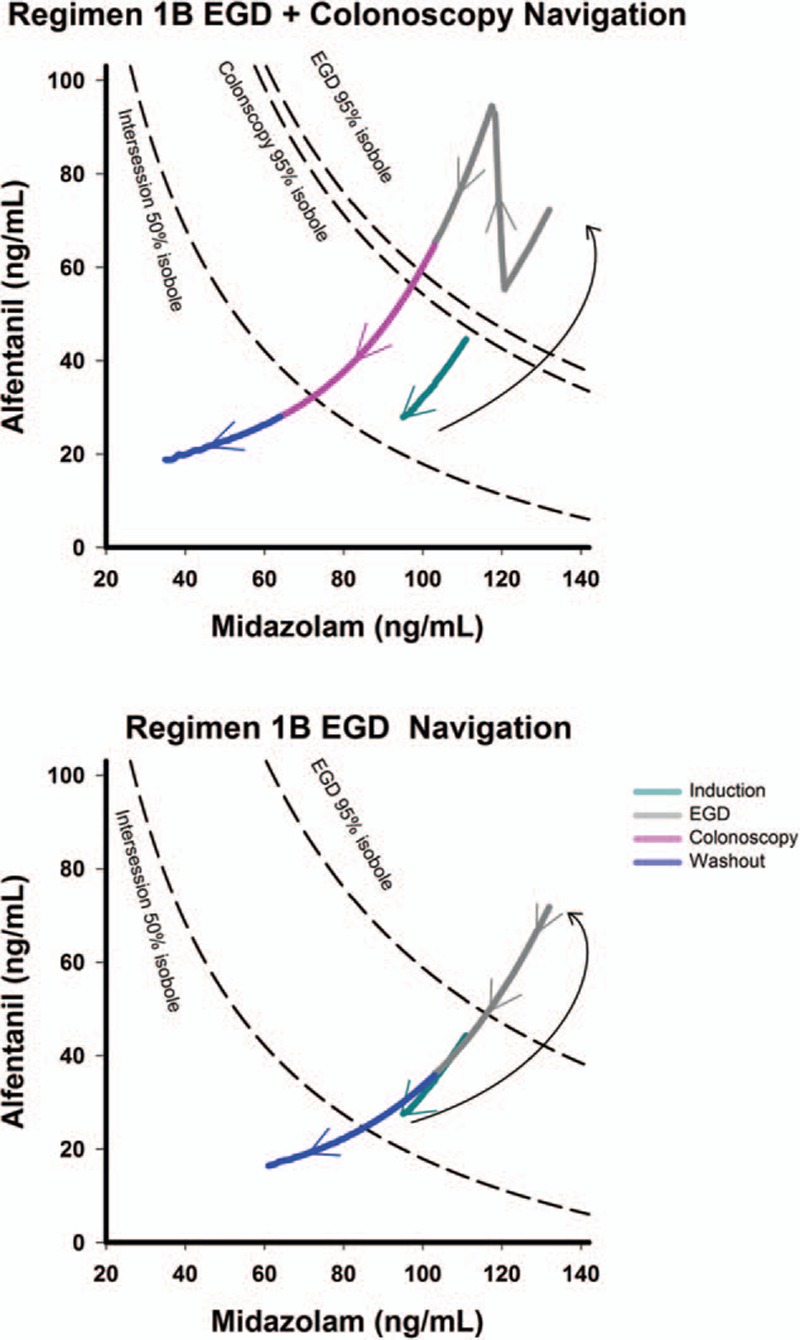
Response surface navigation of regimen 1B. Regimen 1B is the regimen of choice. Response surface is a 3-dimensional graph, but it is less comprehensible for clinical use. We alternatively present the concept using overlapping isoboles derived from 3 response surfaces. The top panel is the simulation of EGD and colonoscopy. The optimal scheme is reaching adequate anesthesia (LOR probability > 95%) and wake up during the washout phase (blue line). The induction dose itself is insufficient to reach LOR for EGD or colonoscopy. Further boluses bring the probability higher and obtaining adequate anesthesia. The bottom panel is the surface navigation during EGD only examination. EGD = esophagogastroduedenoscopy, LOR = loss of response.

We have identified the better performing regimen 1B and the drug dosing scheme is visualized in Figure 5. The 95% isoboles for EGD and colonoscopy are shown and represent adequate anesthetic depth. The intersession 50% isobole marks the defined awakening line. A second bolus of midazolam and alfentanil will bring the probability beyond the 95% isoboles at the start of EGD. A third bolus of alfentanil keeps the EGD phase covered with adequate anesthetic depth. This figure navigates on the response surface and illustrates how the depth of anesthesia changes with different drug boluses.

There are several limitations that merit discussion. First, very high alfentanil or midazolam concentrations were not available in our population used for model building. These concentrations are very rarely used in clinical practice, but if they were to fall in the vicinity, caution should be taken while interpreting the results. Second, during model development, only slightly more than half of the populations were female and have not received prior abdominal surgery. Uncomfortable colonoscopy is known to occur in younger, female, slimmer patients.^42,43^ Our model proposed an average effect and it is expected to underestimate pain in high-risk populations, and overestimates pain in older male patients. It is important to realize that all the pharmacodynamics models tend to oversimplify the complex physiology into simple math equations. No models fit all patients universally and interindividual variations exist. Despite the inherent limitations, RSMs provided extensive assistance in guiding both clinical practice and researches.^44^ Third, we did not analyze the occurrence of hypoxemia or hypotension as a primary endpoint because there were not sufficient amount of data available to build a negative effect RSM. However, we believe that there is a lower rate of hypoxemia with midazolam and alfentanil than propofol.^45^ Fourth, plasma concentrations do not produce the drug effects. The effect-site concentration estimation requires a more sophisticated pharmacokinetic process and the complex drug distribution at a nonsteady state further complicates the accuracy to estimate the effect-site concentrations.^46,47^ Fifth, our study population consists of comparably healthier patients. Sicker patients tend to have altered pharmacokinetic profiles, which could ultimately affect the pharmacodynamic end result. Further studies need to be performed to delineate a safe target for these patients.

In conclusion, we successfully built RSMs to predict patient LOR during gastrointestinal endoscopies. Simulation of regimens with different characteristics gives insights on dosing strategies. A balanced hypnotic-opioid combination consisted of induction dose of 3 mg midazolam and 300 μg alfentanil, followed by 1 mg midazolam/300 μg alfentanil 3 minutes later, and another 300 μg alfentanil 2 minutes later (regimen 1B) performs better in terms of providing adequate anesthesia as well as the most rapid return of consciousness. Although our model can be used by any field of expertise, it is generally accepted that sedation specialists such as anesthesiologists who are airway experts be present while performing sedations. We believe with the aid of our RSM, clinicians can perform sedation with more flexibility and precision.
