# A Response Surface Model Exploration of Dosing Strategies in Gastrointestinal Endoscopies Using Midazolam and Opioids: Erratum

**DOI:** 10.1097/01.md.0000494751.05800.e6

**Published:** 2016-08-26

In the article “A Response Surface Model Exploration of Dosing Strategies in Gastrointestinal Endoscopies Using Midazolam and Opioids”,^1^ which appeared in Volume 95, Issue 23 of *Medicine*, the acknowledgments section was not included in the original article. The article has since been corrected online.
